# Machine learning-based automatic estimation of cortical atrophy using brain computed tomography images

**DOI:** 10.1038/s41598-022-18696-6

**Published:** 2022-08-30

**Authors:** Jae-Won Jang, Jeonghun Kim, Sang-Won Park, Payam Hosseinzadeh Kasani, Yeshin Kim, Seongheon Kim, Soo-Jong Kim, Duk L. Na, Seung Hwan Moon, Sang Won Seo, Joon-Kyung Seong

**Affiliations:** 1 Department of Neurology, School of Medicine, Kangwon National University, Chuncheon, Republic of Korea; 2 Department of Neurology, Kangwon National University Hospital, Chuncheon, Republic of Korea; 3 Department of Medical Bigdata Convergence, Kangwon National University, Chuncheon, Republic of Korea; 4 Department of Biomedical Engineering, Korea University, Seoul, Republic of Korea; 5 Department of Neurology, Samsung Medical Center, Sungkyunkwan University School of Medicine, Seoul, Republic of Korea; 6 Department of Nuclear Medicine, Samsung Medical Center, Sungkyunkwan University School of Medicine, Seoul, Republic of Korea

**Keywords:** Machine learning, Medical research, Biomarkers, Diagnostic markers

## Abstract

Cortical atrophy is measured clinically according to established visual rating scales based on magnetic resonance imaging (MRI). Although brain MRI is the primary imaging marker for neurodegeneration, computed tomography (CT) is also widely used for the early detection and diagnosis of dementia. However, they are seldom investigated. Therefore, we developed a machine learning algorithm for the automatic estimation of cortical atrophy on brain CT. Brain CT images (259 Alzheimer’s dementia and 55 cognitively normal subjects) were visually rated by three neurologists and used for training. We constructed an algorithm by combining the convolutional neural network and regularized logistic regression (RLR). Model performance was then compared with that of neurologists, and feature importance was measured. RLR provided fast and reliable automatic estimations of frontal atrophy (75.2% accuracy, 93.6% sensitivity, 67.2% specificity, and 0.87 area under the curve [AUC]), posterior atrophy (79.6% accuracy, 87.2% sensitivity, 75.9% specificity, and 0.88 AUC), right medial temporal atrophy (81.2% accuracy, 84.7% sensitivity, 79.6% specificity, and 0.88 AUC), and left medial temporal atrophy (77.7% accuracy, 91.1% sensitivity, 72.3% specificity, and 0.90 AUC). We concluded that RLR-based automatic estimation of brain CT provided a comprehensive rating of atrophy that can potentially support physicians in real clinical settings.

## Introduction

Structural brain imaging is recommended in the diagnostic guidelines for dementia^1,2^, and it is known to be related to cognitive dysfunction in normal elderly or patients with Alzheimer's disease (AD)^3,4^. In addition to excluding surgical lesions, the assessment of cerebral atrophy suggestive of underlying pathology, including the medial temporal lobe for AD^5^, is possible with the use of structural brain imaging. Several visual rating scales based on structural imaging have been reported to assess brain atrophy, with some of them being widely used in research and clinical practice^5,6^. Compared to quantitative volumetric measures, visual rating scales have the advantage of directly applying clinically acquired images without a time-consuming process^7^. However, there are reliability issues for inter- or intra-rater agreement^8,9^, and most of the visual rating scales are made based on brain magnetic resonance imaging (MRI)^7^. Although brain MRI is the primary imaging marker for neurodegeneration according to the recent research framework for AD^10^, brain computed tomography (CT) may also yield important information in subjects with dementia^11^. More importantly, brain CT is increasingly being used for the early screening of dementia, as a part of the Korean national dementia plan since 2008^12^. Therefore, there is a growing need for a reliable and practical method for brain CT visual rating, which was seldom investigated.

Computerized, automated grading of brain CT has potential benefits for maximizing reliability and efficiency, resulting in the coverage of a screening system for cognitive decline. The algorithm for visual rating is necessary to increase the clinical utility of automated grading. This suggests that an “artificial intelligence (AI)–based, computer-aided (CAD)” approach will be needed for structural imaging-based visual rating scales based to discover and analyze neurodegeneration by enabling the statistical power to detect subtle effects.

AI is an all-embracing field of computer science that use computers and machines to perform certain tasks to mimic the problem-solving and decision-making capabilities of the human mind, thereby creating an intelligent system. Machine learning, representation learning, and deep learning are subfields of AI. A growing number of clinical applications based on machine learning and deep learning have been proposed in neuroimaging for risk assessment, diagnosis, prognosis, and prediction^13^. Therefore, machine learning, which adopts explicit features specified by experts and has shown potential benefits in identifying dementia risk on neuroimaging^14^, could be used for the automated grading of brain CT to support clinical assessment.

As such, this study aimed to develop machine learning algorithms for the automatic grading of cortical atrophy using brain CT and compare these with the visual ratings of neurologists.

## Materials and methods

### Subjects

We recruited 259 patients with AD and 55 cognitively normal (CN) subjects, who underwent brain MRI and CT. Alzheimer’s disease was diagnosed based on the National Institute on Aging-Alzheimer's Association (NIA-AA) research criteria for probable AD^1^. Subjects with normal cognition were defined as those without any history of neurologic or psychiatric disorders, and normal cognitive function was determined using neuropsychological tests.

All subjects were evaluated by clinical interview, neurological examination, neuropsychological tests, and laboratory tests, including complete blood count, blood chemistry, vitamin B12/folate, syphilis serology, and thyroid function tests. Brain MRI confirmed the absence of structural lesions, including territorial cerebral infarctions, brain tumors, hippocampal sclerosis, and vascular malformations. Demographic data are described in Table 1. The study included 55 participants with normal cognition (NC) and 256 participants with AD. The mean age (standard deviation) of participants with NC was 53.1 (20.2), while that of participants with AD was 69.0 (10.4). Women comprised 28 participants (50.9%) in those with NC and 146 participants (56.4%) in those with AD.

**Table 1:** Demographics of the study participants.

	CN (n = 55)	AD (n = 259)	p-value
Age(years)	53.1 (20.2)	69.0 (10.4)	< 0.001
Sex(F)	28 (50.9)	146 (56.4)	0.459
Education (years)	14.5 (2.7)	11.8 (4.8)	0.002
MMSE	28.8 (0.9)	18.6 (5.7)	< 0.001

Values are mean (SD) or N (%). Statistical analyses were performed with Chi-square or 'Student's *t*-tests.

*SD* Standard deviation, *CN* Cognitively normal, *AD* Alzheimer’s disease, *MMSE* Mini-mental state examination.

This study protocol was approved by the Institutional Review Board of Samsung Medical Center (approval No. 2017-07-039). We obtained written informed consent from each subject, and all procedures were carried out according to the approved guidelines.

### Image acquisition

We acquired standardized, three-dimensional, T1 turbo field echo images from all subjects at Samsung Medical Center using the same 3.0 T MRI scanner (Philips Achieva; Philips Healthcare, Andover, MA, USA) with the following parameters: sagittal slice thickness of 1.0 mm, over contiguous slices with 50% overlap, no gap, repetition time (TR) of 9.9 ms, echo time (TE) of 4.6 ms, flip angle of 8°, and matrix size of 240 × 240 pixels, reconstructed to 480 × 480 over a field of view of 240 mm. So, real scanner resolution of MR images has isotropic 1.0 mm voxel size and voxel size of our reconstructed T1 MR images are isotropic 0.5 mm voxel size by overlapping between contiguous slices.

We acquired CT images from all subjects at Samsung Medical Center using a Discovery STe PET/CT scanner (GE Medical Systems, Milwaukee, WI, USA) in the three-dimensional scanning mode, which examines 47, 3.3-mm thick slices spanning the entire brain^15,16^. CT images were also acquired using a 16-slice helical CT (140 keV, 80 mA, 3.75-mm section width) for attenuation correction. Voxel size of CT images acquired by PET-CT scanner are 0.5 mm × 0.5 mm × 3.27 mm. The SNR value was checked through Phantom study (3.75 mm slice thickness, 120 kVp, 190 mA), and it was conducted by GE Discovery STe PET-CT scanner. The SNR results of our phantom study were Water Layer: 0.23 ± 0.04, Acrylic Layer: 25.97 ± 1.34, respectively. The SNR was calculated as “SNR = CT Number / SD (noise).

### Preprocessing

The two different modalities of brain imaging underwent preprocessing before using the segmentation network. First, the CT images in Digital Imaging and Communications in Medicine (DICOM) data format were converted to Neuroimaging Informatics Technology Initiative (NIFTI) data format by applying dcm2niix (Chris Rorden's dcm2niiX version v1.0.20171017 [OpenJPEG build] GCC4.4.7 [64-bit Linux]). Then, we aligned the 3D CT images to the corresponding T1 MR images using the FMRIB's Linear Image Registration Tool (FLIRT), a tool of the FSL software (https://fsl.fmrib.ox.ac.uk/fsl/fslwiki; FMRIB Software Library v6.0)^17,18^. Image registration was conducted using rigid body affine transformation. The registration process is fully automatic with parameters of both 6 degrees of freedom and spline interpolation methods.

The Brain Extraction Tool (BET)^19^, another tool of the FSL software, was also a validated means for skull stripping in brain CT images^20^. After setting up each image to the general brain tissue range, we used BET with a fractional intensity (FI) value of equal to 0.01, acquiring the skull-stripped CT images. Every skull-stripped CT image was manually checked to assure clear results. Afterwards, intensity normalization and histogram equalization were applied to each image. Finally, we downsized the CT slices to 128 × 128 pixels on account of GPU performance.

Following preprocessing of CT images, T1 MR images were employed for training the deep learning-based CT image segmentation model. To obtain pre-defined anatomical labels, MR images were preprocessed using the FreeSurfer software (https://surfer.nmr.mgh.harvard.edu)^21,22^. Considering low contrast in CT images, the automatic segmentation of CT images was done only for three parcellations: cerebrospinal fluid (CSF), white matter (WM), and gray matter (GM). MR slices were also downsized to half their pixel for the same reason above.

### Segmentation

**Figure 1:**
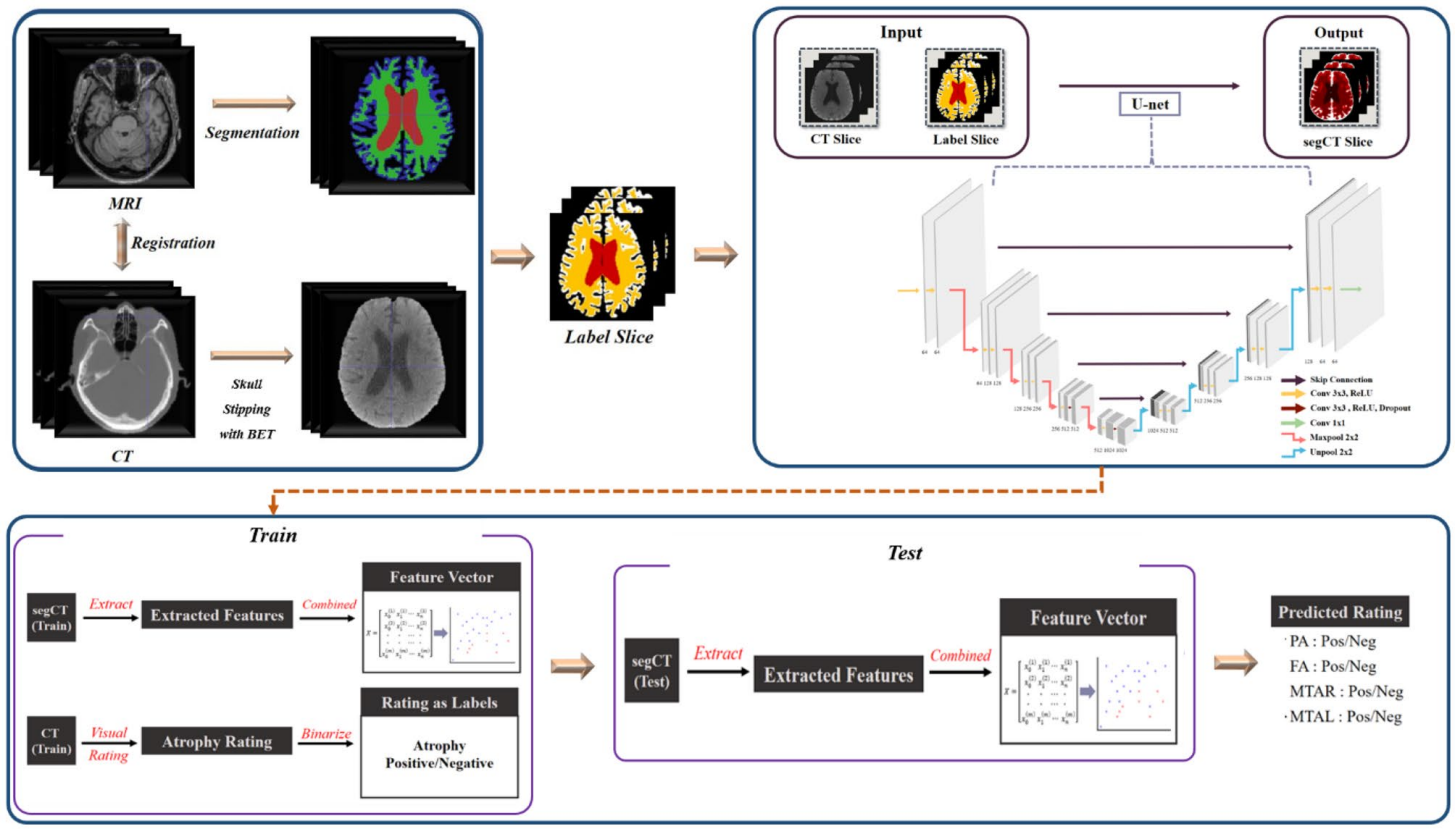
A scheme of framework. Tensors are indicated as boxes while arrows denote computational operations. Number of channers is indicated beneath each box. Input and output of this network are CT slice with Label slice pairs and segmented CT images (segCT) slice. The classification conducted by threefold cross-validation was performed by randomly assigned the subject into three subgroups. *BET* Brain extraction, *ReRU* rectified linear unit activation, *segCT* Segmented CT, *RLR* regularized logistic regression, *FA* frontal atrophy, *PA* Parietal atrophy, *MTAR* medial temporal atrophy, right, *MTAL* medial temporal atrophy, left, *Pos* positive, *Neg* negative.

A convolutional neural network (CNN) was implemented to attain the segmentation image of brain CT. Among the diverse, state-of-the-art networks, we chose the 2D form of U-Net, which has already been proven to be effective in various biomedical segmentation studies^23^. Preprocessed 3D CT images were sliced into 2D axial slices, and brain-invisible slices were removed beforehand. Similarly, segmented 3D MR images were sliced in the same manner. We then attempted to build a CNN segmentation network using the refined 2D CT image as an input and simultaneously commensurate MR as an answer label. Figure 1 illustrates the schematic diagram of the U-Net framework. Segmentation outputs were obtained through the U-Net architecture with 10 different model weights via a ten-fold train/test split. During the training process, we fine-tuned each resulting model using an adaptive moment estimation (Adam) optimizer, with 1e-6 as the initial learning rate and 16 as the batch size. We also applied an early stopping method to prevent overfitting. The quality of every segmentation result was manually checked, and for further validation, we computed the dice similarity coefficient (DSC), which is defined as

$${\text{DSC}} = \frac{{2\left| {X \cap Y} \right|}}{\left| X \right| + \left| Y \right|}$$

X represents the answer slice region, Y represents the result slice region.

Unfit images due to subject overlap, conversion failure, registration mismatch, and visual rating error were removed after each preprocessing stage, leaving a total of 314 subjects for analysis.

### Visual rating of cortical atrophy

The visual rating of cortical atrophy was performed for the frontal lobe, parietal lobe, and medial temporal lobe by rating the brain CT axial images. Frontal atrophy (FA) was assessed using the simplified Pasquier scale or global cortical atrophy for the frontal lobe (GCA-F)^8^. Parietal atrophy (PA) was measured using the axial template of the posterior atrophy scale^24^. Lastly, medial temporal atrophy (MTA) was assessed using the hippocampus and surrounding CSF^25^, showing good agreement with Scheltens' coronal visual rating scale^5^. FA and PA were evaluated as four-point scales (from 0 to 3), whereas MTA was evaluated using a five-point scale (from 0 to 4). More severe atrophies were measured when there was asymmetry for FA and PA, while bilateral atrophies were separately measured for MTA. The visual rating was performed by three neurologists (Jae-Won Jang, Seongheon Kim, and Yeshin Kim), who were blinded to demographic and clinical information. If there were discrepancies among them, a consensus was made after reviewing the cases. The inter-rater and intra-rater reliability with randomly selected brain CT images from 20% of all images were deemed excellent, with values of 0.81 ~ 0.92 and 0.90 ~ 0.94, respectively (Supplementary Table 1).

### Feature extraction

The ratio of GM, WM, and ventricular size was extracted from the segmented CT images, serving as features for the classification step. Given the difficulty of extracting PA, FA, and MTA from CT, we attempted to use the most typical atrophy in AD diagnosis, which is global cortical atrophy (GCA). We respectively drew out features in the 3D volume and 2D slice, which are all related to GCA on a certain level. A detailed description of the extracted features is provided below:

- The volume ratio: GMR3D (the volume ratio of GM), WMR3D (the volume ratio of WM), GMWMR3D (the volume ratio of the sum of GM and WM in the whole-brain volume), Ven3D (the total voxel number of the ventricle in the whole-brain volume).
- The area of ratio: GMR2D (the area ratio of GM), WMR2D (the area ratio of WM), GMWMR2D (the area ratio of the sum of GM and WM in the particular brain slice, which indicates the visible slice prior to the ventricle when looking at the brain from top to bottom in the axial orientation), Ven2D (the total pixel number of the ventricle in the particular brain slice, which indicates the slice with the best-observed ventricle shown as a "butterfly" shape).

### Atrophy classification

The consolidated pipeline of the classification framework is provided in Fig. 1. Prior to full-scale classification, we cut off the current atrophy rating score with multiple degrees to two degrees—positive and negative. For example, regarding FA and PA, visual rating scale (VRS) scores of 0 and 1 were considered negative, whereas VRS scores of 2 and 3 were considered positive. Similarly, regarding MTAs, VRS scores of 0 and 1 were considered as negative, whereas VRS scores of 2, 3, and 4 were considered as positive.

For the classification phase, we opted to use one of the simplest supervised learning algorithms with a high bias, a regularized logistic regression (RLR), considering the data status and the number of extracted features. Logistic regression has also been widely recognized for AD-related classification works in neuroimaging^26^. The RLR model is trained using the extracted features and makes the final prediction regarding atrophy diagnosis in different brain positions. Major hyperparameters (e.g., penalization type, regularization strength, optimization solver algorithm) were tuned by the random search method in threefold cross validation within a pre-set range. The process was thoroughly handled using the Scikit-learn software, a python machine learning package^27^. Afterwards, we plotted a receiver operating characteristic curve (ROC curve) for the performance evaluation and calculated the area under the curve (AUC score). We then selected the optimal cut-off point with the maximum value of Youden's index, which is defined as

$${\text{Youden's }}\,{\text{Index }}\;{\text{J}} = {\text{Sensitivity}} + {\text{Specificity}} - 1$$

Several metrics, including sensitivity (SENS), specificity (SPEC), and classification accuracy (ACC), were also calculated at this point.

## Results

### CT segmentation

**Figure 2:**
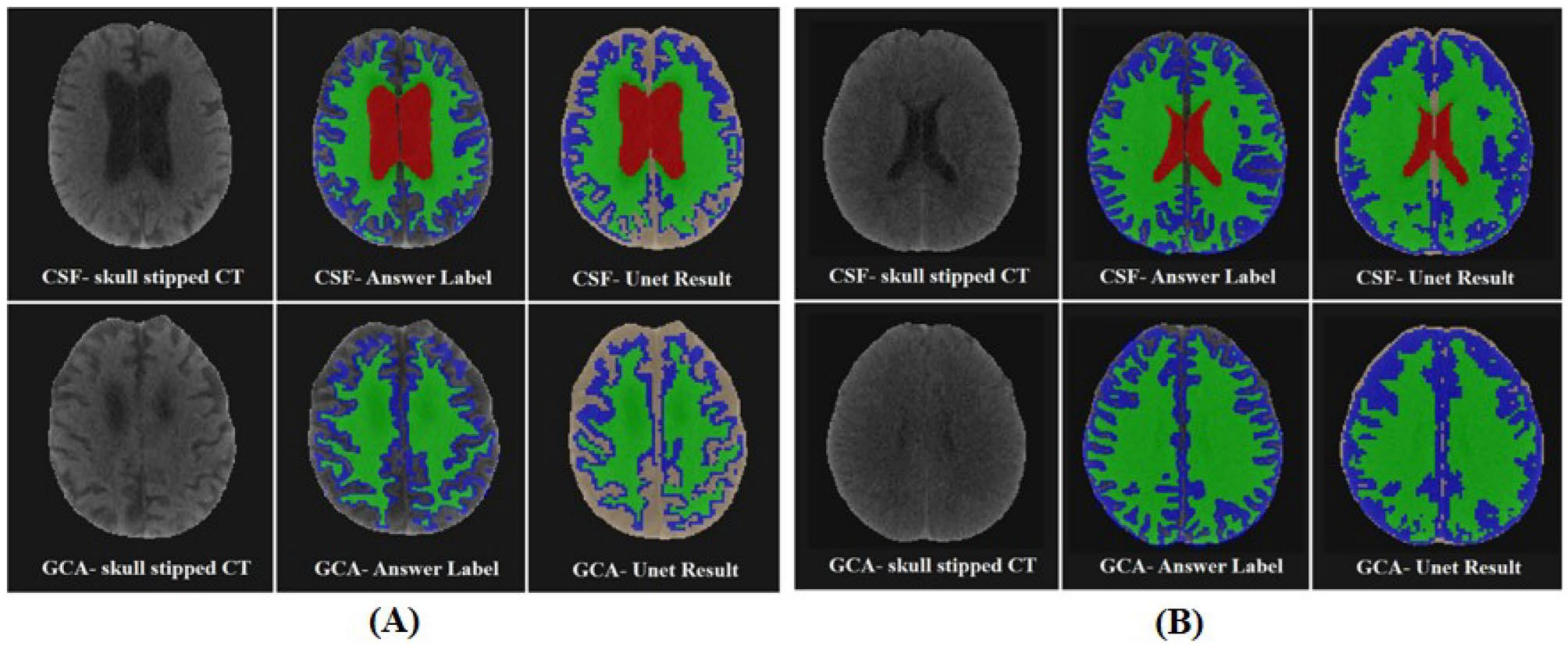
Sample of segmentation result (**A**) Alzheimer’s dementia (**B**) Normal control. *GCA* global cortical atrophy, *CSF* cerebrospinal fluid. Green = white matter, blue = gray matter, red = CSF.

In Fig. 2, CT segmentation results from the U-Net concerning both participants with AD and NC are illustrated. The presented slices, referred to as CSF and GCA, were closely related to AD diagnosis. The DSC was calculated for every subject, and its average was 0.725 ± 0.0443. Although the result is not at the highest level, it was not a major barrier to the final classification phase. Furthermore, CT segmentation had turned out to be the way we demanded, which was the rough parcellation of the brain into CSF, WM, and GM.

### Classification performance

**Figure 3:**
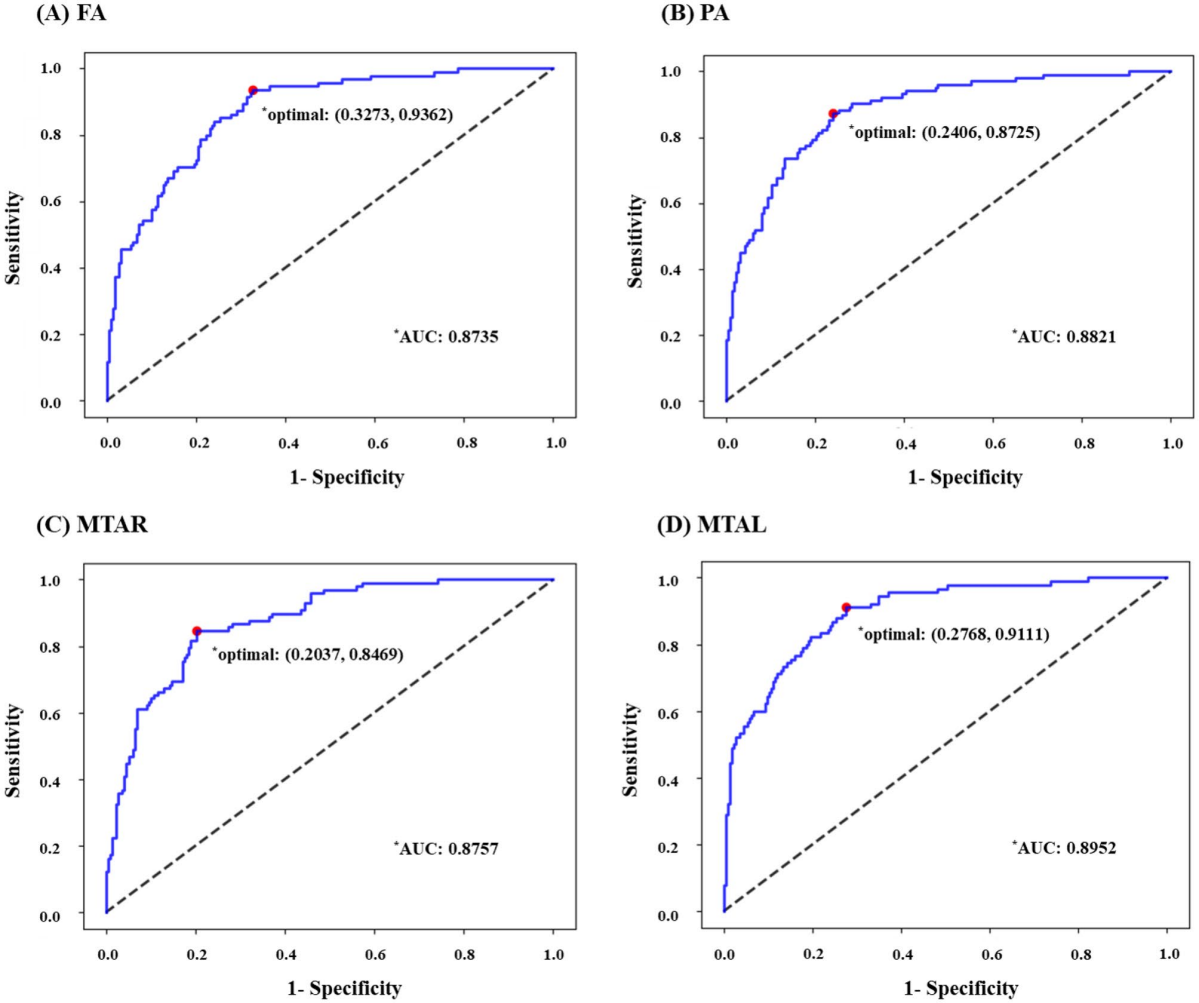
ROC Curve for binary classification of atrophy. *FA* frontal atrophy, *PA* Parietal atrophy, *MTAR* medial temporal atrophy, right, *MTAL* medial temporal atrophy, left.

As shown in Table 2 and Fig. 3 the proposed RLR model achieved an AUC score of 0.8735 in FA, 0.8821 in PA, 0.8757 in MTAR, and 0.8952 in MTAL. At the maximum value of Youden’s Index in each case, the model showed sensitivities of 93.52% in FA, 87.25% in PA, 84.69% in MTAR, and 89.52% in MTAL; specificities of 67.27% in FA, 75.94% in PA, 79.63% in MTAR, and 72.32% in MTAL; and accuracies of 75.16% in FA, 79.62% in PA, 81.21% in MTAR, and 77.71%. Among all four regions, MTAL performed the best in terms of the AUC rate, whereas PA had the AUC lowest rate. In terms of sensitivity, although PA had the lowest SPE and AUC rates, it achieved the greatest rate in terms of SENS. Moreover, MTAR demonstrated the highest value for SPEC and AUCC.

**Table 2:** Binary classification result of atrophy rating through regularized logistic regression.

Atrophy type	AUC	At maximum value of Youden’s Index for the ROC Curve		
		SENS	SPEC	ACC
FA	0.8735	0.9362	0.6727	0.7516
PA	0.8821	0.8725	0.7594	0.7962
MTAR	0.8757	0.8469	0.7963	0.8121
MTAL	0.8952	0.9111	0.7232	0.7771

*FA* frontal atrophy, *PA* Parietal atrophy, *MTAR* medial temporal atrophy, right, *MTAL* medial temporal atrophy, left.

### Analysis on the importance of extracted features

**Figure 4:**
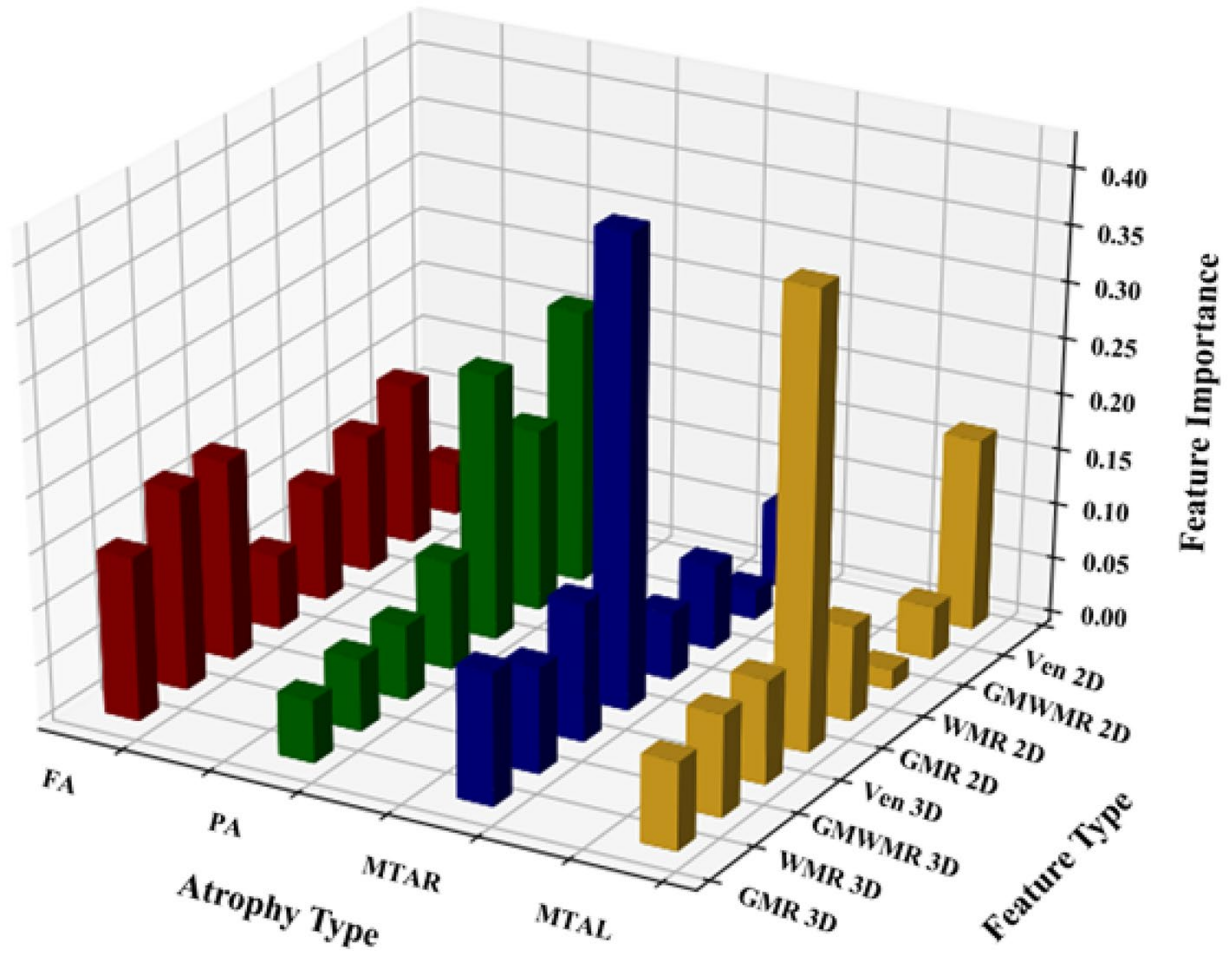
Feature importance of visual rating scale. *FA* frontal atrophy; *PA* parietal atrophy; *MTAR* medial temporal atrophy, right; *MTAL* medial temporal atrophy, left; *Ven2D* (the area ratio of ventricle); *GMWMR2D* (the area ratio of the sum of gray matter [GM] and white matter [WM]); *WMR2D* (the area ratio of WM); GMR2D (the area ratio of GM); Ven3D (the volume ratio of the ventricle); *GMWMR3D* (the volume ratio of the sum of GM and WM); *WMR3D* (the volume ratio of WM); GMR3D (the volume ratio of GM).

We further investigated how the extracted features utilized to train the classification model affected the decision-making process for each atrophy case. In the trained RLR models, we normalized the value of feature coefficients congruous to each feature, visualizing them as a 3D bar plot (Fig. 4). The magnitude of the feature coefficients for eight features (in the order of GMR3D, WMR3D, GMWMR3D, Ven3D, GMR2D, WMR2D, GMWMR2D and Ven2D) were 0.148, 0.182, 0.181, 0.0680, 0.104, 0.124, 0.146, and 0.0477 in FA; 0.0572, 0.0658, 0.0677, 0.0982, 0.240, 0.165, 0.247, and 0.0598 in PA; 0.120, 0.0958, 0.125, 0.421, 0.0604, 0.0766, 0.0266, and 0.0741 in MTAR; and 0.0801, 0.0928, 0.0948, 0.407, 0.0861, 0.0181, 0.0480, and 0.173 in MTAL. Results revealed that larger coefficient means showed greater influence on final decision making.

FA classification was influenced by across-the-board GCA features from both 3D volume and 2D slice. In the case of PA, GCA features from a particular 2D slice greatly affected classification. Meanwhile, MTAs could be classified according to ventricular changes in both 3D volume and 2D slice, rather than GCA features as in the previous cases.

## Discussion

A machine learning-based automated atrophy estimator was developed to aid clinicians in deciding whether there was significant atrophy in brain CT. In this study, the proposed RLR model showed good agreement with the visual rating scale and high reliability.

Since the Korean national dementia plan started in 2008, approximately 30,000 brain CT scans are being taken nationwide annually for the differential diagnosis of dementia, and this number is gradually increasing^12^. Despite the abundance of brain CT data being accumulated, there has been an unmet need for visual rating due to low resolution in images and low reliability in evaluators.

Although automated CT-based segmentation and total intracranial volume quantification have been previously reported^28^, there are similar shortcomings of automated volumetric analysis with brain MRI. In other words, brain MRI visual rating requires expertise that cannot be applied in clinical practice for individual subjects directly^7^. In contrast, once the algorithm for brain CT has been set up, the machine learning-based automated atrophy rating can be easily applied to identify atrophy on behalf of the evaluator. Therefore, this method can be used not only for the direct assessment of atrophy in individual patients, but also to consistently reproduce approximate expert levels of visual ratings in a large number of brain CT scans. This is significant as the manual segmentation of the pixels, labeling, and region of interest in an image require experienced readers, is time-consuming, and is prone to intra-and inter-observer variability.

Over the past years, several automatic and semi-automatic brain segmentation methods have been proposed for both MRI and CT imaging^29–31^. The small and complex structure of the brain makes segmentation a challenging issue, especially in CT scans due to much lower SNR and lower spatial resolution as major limitations. Image segmentation can be used for a better visualization of the morphological changes of brain atrophy, providing the clinician with a better understanding and assisting them in the diagnostic process. In this study, our proposed methodology proved to be efficient and effective on CT-based imaging and can be easily leveraged for similar automated brain atrophy grading applications on different imaging modalities.

Although downsizing the matrix of initial acquisition images was performed accounting for GPU performance, the process was rapid as a few seconds and effective in getting results that could support clinical decisions. Moreover, we tried to reduce noise for brain segmentation and efficient quantitative performance effects through the preprocessing phase, which was done with the intensity and histogram normalization of CT images and affine transformation by using MRI images of individual subjects. Therefore, it is possible to provide consistent results by providing segmented tissue types of GM, CSF, and WM in MRI and CT images.

While the accuracy and reliability of the RLR measures were high for all groups, MTAR achieved the highest accuracy rate compared to the MTAL, PA, and FA regions. Contrarily, the FA region was the lowest in three out of the four evaluation metrics, which was possibly due to the relative obscurity of FA in AD as compared to MA or PA. The proposed RLR model also revealed an accuracy of 80%, since approximately 20% did not fit the visual rating of the neurologists. This may be due to the ambiguity of the visual rating of brain CT itself. Furthermore, the visual rating was used as a labeling value for machine learning training; however, atrophy rating based on brain CT, which showed a relatively low resolution compared to brain MRI, was not easy for a neurologist to identify. As our goal was not to measure accurate brain atrophy but to develop algorithms that can more consistently predict atrophy to aid clinical decisions, we believe the more labeled data could enhance accuracy.

Feature contribution and its visualization is an important analysis method in the field of machine learning. To identify which atrophy-related region contributed most to the performance of the proposed models, we evaluated feature importance based on the coefficient value of each feature. Among the highest ranked features, GCA features from both 3D volume and 2D slice were the most important for FA classification, whereas GCA features from only a unique 2D slice greatly affected PA classification. Our proposed model also showed that ventricular changes in both 3D volume and 2D slice significantly contributed to the classification of MTAs among their other counterparts.

Despite the promising results, the study had limitations. First, the small available training dataset may have affected model performance. It is reasonable to expect that a larger CT-based dataset would improve the accuracy of the method. In comparison to the MRI-based dataset, it is worth mentioning that MRI images provide significantly more detail than the CT-based scans, which have a more uniform feature distribution. This may further support the idea that having extra CT-based data for model training will enhance the accuracy rate for brain atrophy estimation. As such, exploring deep learning strategies that can generate synthetic medical images, such as data augmentation techniques, may be required. Second, a monocentric dataset has been applied in this study. All CT scans were acquired at a single institution, and our models did not account for variations in hardware implementation and scanning techniques across institutions. This may bias the results and consequently reduce the generalizability of the trained CNN model. Also, SNR values might be changed depending on equipment (slice thickness), protocol (mAs), measurement target or patient (size or organ), so it is difficult to talk about it uniformly, but it can be used to show the CT scanner status as SNR of our phantom study. However, the availability of large, diverse, annotated datasets for training remains a considerable challenge in developing the CNN models. Third, CT images are involved with high radiation exposure to patients. Therefore, they cannot be routinely recommended in clinical practice despite of advantages over MRI in terms of price and accessibility. This study tried to present a useful model for the evaluation of atrophy in brain CT, which has been steadily accumulated as government-led early diagnosis program in Korean dementia plan.

In conclusion, an integrated U-Net model for brain atrophy segmentation with an RLR model provided a comprehensive clinical tool for the visual rating of brain atrophy. These measures may increase diagnostic and predictive information to better evaluate a wide variety of regions in brain atrophy conditions, especially in hospitals with limited resources. Moreover, collecting large-scale data sets can help accelerate CT-based rating application in clinical medicine, and there is potential to improve these machine learning-based methods. Further studies should aim to expand this tool to assist neurologists in real clinical settings.

## Supplementary Information

Supplementary Information.

## Data Availability

The datasets used and/or analysed during the current study available from the corresponding author on reasonable request.
