# Supplementary material for: Machine learning-based automatic estimation of cortical atrophy using brain computed tomography images
Source: Sci Rep. 2022 Aug 30;12:14740. doi: 10.1038/s41598-022-18696-6 (PMC9427760; doi:10.1038/s41598-022-18696-6)
Supplement: Supplementary file 1 — Supplementary Information. [file 41598_2022_18696_MOESM1_ESM.docx]

**Supplementary Materials**

Supplement table 1. Values for inter-rater and inter-rate agreement of visual rating scales.

|  | Inter-rater (95%CI) | Intra-rater (95%CI) |
| --- | --- | --- |
| Frontal atrophy | 0.82 (0.74-0.89) | 0.90 (0.83-0.94) |
| Parietal atrophy | 0.81 (0.71-0.88) | 0.94 (0.92-0.96) |
| Medial temporal atrophy(right) | 0.90 (0.84-0.93) | 0.91 (0.86-0.95) |
| Medial temporal atrophy(left) | 0.92 (0.88-0.95) | 0.91 (0.86-0.95) |
